# Isolation and Identification of *Legionella* spp. in environmental water sources based on macrophage infectivity potentiator (*mip*) gene sequencing in southwest Iran

**DOI:** 10.3934/microbiol.2019.3.223

**Published:** 2019-08-16

**Authors:** Mojtaba Moosavian, Mina Moradzadeh, Ataollah Ghadiri, Morteza Saki

**Affiliations:** 1 Infectious and Tropical Diseases Research Center, Health Research Institute, Ahvaz Jundishapur University of Medical Sciences, Ahvaz, Iran; 2 Department of Microbiology, Faculty of Medicine, Ahvaz Jundishapur University of Medical Sciences, Ahvaz, Iran; 3 Department of Immunology, Faculty of Medicine, Ahvaz Jundishapur University of Medical Sciences, Ahvaz, Iran; 4 Cellular and Molecular Research Center, Ahvaz Jundishapur University of Medical Sciences, Ahvaz, Iran; 5 Student Research Committee, Ahvaz Jundishapur University of Medical Sciences, Ahvaz, Iran

**Keywords:** *Legionella*, *mip* gene, macrophage infectivity potentiator, water supplies

## Abstract

*Legionella* species are widespread in natural water sources and man-made aqueous environments, as well as fresh-water. The present study was conducted owing to the lack of research regarding the prevalence of *Legionella* spp in the water sources of Ahvaz city in southwest Iran. In this study the macrophage infectivity potentiator (*mip*) gene sequencing was used for identification of various *Legionella* species isolated from different water sources. In this study, 144 water samples were collected and inoculated on the buffered charcoal-yeast extract (BCYE) agar and modified Wadowsky-Yee (MWY) medium. The DNA was extracted from positive cultures. The *Legionella* species were confirmed by amplifying a 654 bp fragment of the *16S rRNA* gene. The *mip* gene of all isolates were amplified by PCR and purified for sequencing. The *mip* gene sequences were analyzed by jPHYDIT software version 1. The results showed a 13.9% (20/144) prevalence of *Legionella* spp. in water sources of Ahvaz city, southwest Iran. Analyzing of the mip gene sequences showed, out of 20 *Legionella* isolates, 13 isolates (54.1%) were positive for L. pneumophila, 5 isolates (20.8%) were positive for L. *worsleinsis*, one isolates for each one of L. dumoffi and L. fairfieldensis, (4.1%). According to our research, the occurrence of *Legionella* spp in water sources could be a hazard for the health systems especially in the hospitals. The regular monitoring of these water sources by health planners may therefore be useful for decreasing the risk for *Legionella* spp. infections.

## 1. Introduction

*Legionella* species are widespread in natural water sources and man-made aqueous environments, as well as in fresh-water and wet soils [1]. These bacteria spread by *Legionella*-containing water aerosols and cause infection in exposed people who have risk factors such as smoking, age more than 50 years, weakened immune system, diabetes, cancer, or chronic lung disease [2],[3]. In most cases, Legionnaires' disease (LD), the sever pneumonic form of disease, is caused by *Legionella pneumophila* serogroup 1 (*L. pneumophila* SG-1), while the Pontiac fever, milder form of the disease, occurs by other non-*pneumophila* *Legionella* species [3]. The presence of *Legionella* spp. is favored by the attendance of algae and protozoa (amoebas and ciliates), in which they multiply intracellularly [4].

*Legionella* spp. have increased tolerance to chlorination, so they could enter potable hot water systems and multiply in different water sources, including cooling towers, whirlpool spas, hot tubes ,fishponds, shower heads, , holding tanks and respiratory ventilators [1],[5]. Although some of studies have indicated that remaining more than 2 mg/l of chlorine in water, could eradicate free-living *Legionella* [6], however, *L. pneumophila* living within biofilms is more resistant to annihilation through chlorine [7].

Although the culture method remains among the detection tools of *Legionella* in water sources, the excessive growth of accompanying living organisms or the conversion of *Legionella* cells to a viable but nonculturable form (VBNC) may affect the culture's results [8],[9]. Thus, an effective assay for detection of *Legionella* species appears to be essential. It should be emphasized that detection of *Legionella* on the genus level as well as differentiation between *L. pneumophila* and non-*L. pneumophila* species is highly important for hazard prediction and the elimination of *Legionella* [8],[10].

So far, several molecular methods that target *Legionella* spp. in water sources, have been reported, including *L. pneumophila* PAL antigen, and the polymerase chain reaction (PCR) assays targeting the *5S rRNA* gene, the 23S-5S spacer region, the *16S rRNA* gene, and the macrophage infectivity potentiator (*mip*) gene [8],[11]–[13]. Among the aforementioned methods, PCR amplification and sequencing of the *mip* gene seems to be a reliable method, which has known as a standard method [14]. In this field, the database, established by the members of the European Working Group for *Legionella* Infections (EWGLI), is freely accessible, which contains the data from all valid described species [15].

The present study aimed to evaluate the prevalence of *Legionella* spp. in water samples and to determine the species by mip gene sequencing in Ahvaz, southwest Iran.

## 2. Materials and methods

### 2.1. Ethical Consideration

Not applicable.

### 2.2. Collection, preparation, and culture of water samples

In this study, 144 water samples were collected from different water supplies in 500 mL sterile plastic containers without neutralizing agent, from May to July 2014. Before sampling, the water sample was mixed with the sediment. The water samples were taken from the 5 teaching hospitals of Ahvaz Jundishapur University of Medical Sciences, Ahvaz, Iran. However, some of the water sources were located out of hospital environments.

In the laboratory, each water sample was centrifuged at 3000 g for 30 min at 25 °C. The 0.5 mL of sediment was treated by 4.5 mL washing buffer acid (HCL/KCL, pH 2.2) for 4 min. After mixing by wortex, 200 µL of the suspension was inoculated on two non-selective and selective media, buffered charcoal-yeast extract (BCYE) agar, and modified Wadowsky-Yee (MWY) medium (Oxoid, UK), respectively. The plates were incubated at 37 °C in a 5% CO_2_ humidified jar for 7–14 days. These plates were monitored at least three times for growth of colonies (first time: after 3 or 4 days) until the end of the incubation period. The colonies with typical *Legionella* morphology were sub-cultured on BCYE agar with and without L-cysteine, blood agar and MacConkey agar plates. In case which the isolate could grow on BCYE agar with L. cysteine, but not on the other media, and stained as a gram-negative rod, it was considered as *Legionella* spp. [16]. In this study, *L. pneumophila* ATCC 33152 was used as a quality control.

### 2.3. DNA extraction

DNA extraction was performed using the boiling method. In this method, a few colonies were dissolved in Tris-EDTA (TE) buffer (10mM Tris, 0.1mM EDTA, pH 8.0). The suspension was incubated in a dry-block thermostat (Biosan, Latvia) at a temperature of 99 °C for 10 min and then, placed in an ice box at a temperature of −20 °C for 5 min. The suspension was subsequently centrifuged at 14000 rpm for 5 min and the supernatant was transferred to a new micro tube and kept at −20 °C for molecular assay [17]. The concentration and quality of DNA were evaluated by measuring the absorbance of A260 and A280 nm with a spectrophotometer (Thermo Fisher Scientific, USA) and agarose gel electrophoresis, respectively.

### 2.4. PCR assay of 16S rRNA and mip gene

The *Legionella 16S rRNA* gene was amplified in the thermocycler (Eppendorf, Germany) using a primer pair including forward 5′-AAGATTAGCCTGCGTCCGAT-3′ and reverse 5′-GTCAACTTATCGCGTTTGCT-3′as described previously [17]. PCR master mix was prepared in a final volume of 25 µL containing 10 X PCR Buffer (2.5 µL), MgCl2 50 mM (0.75 µL), dNTPs 10 mM (0.5 µL), each primer 10 µM (1 µL), Taq DNA Polymerase 5 U/µL (0.25 µl), Extracted DNA 500ng (5 µL) and 14 µl of distilled water. DNA amplification was performed in a thermocycler (Eppendorf, Germany) under conditions of the pre-denaturation at 94 °C for 5 min, followed by 30 cycles at 94, 54, and 72 °C (each for 1 min), and a final extension at 72 °C for 10 min. In each PCR run, *L. pneumophila* ATCC 33152, and sterile distilled water were used as the positive and negative controls, respectively. A second PCR assay was carried out for the identification of *Legionella* species by DNA sequencing, targeting the *mip* gene (661–715 bp) of *Legionella* spp. [14]. The primer sequences were as follows (with parentheses indicating a mixed-base site): forward primer (Legmip-f), 5′-GGG(AG)ATT(ACG)TTTATGAAGA TGA(AG)A(CT)TGG-3′; reverse primer (Legmip-r), 5′-TC(AG)TT(ATCG)GG(ATG)CC (ATG)AT(ATCG)GG(ATCG)CC(ATG)CC-3′. Gene amplification was carried out in following conditions: the pre-denaturation at 94 °C for 5 min, 35 cycles of denaturation at 95 °C for 1 min, annealing for 2 min at 57°C, and extension for 2 min at 72 °C and a final extension at 72 °C for 10 min.

### 2.5. Gel electrophoresis and mip gene sequencing

The PCR products were loaded and separated onto a 2% agarose gel prepared in 1 X TAE (Tris/Acetate/EDTA) buffer. The amplicons were visualized using ultraviolet light gel documentation system (Protein Simple, San Jose, CA, USA) after staining with ethidium bromide 0.5 µg/mL (Cinnaclone, Tehran, Iran). A 100 bp DNA ladder was used as a size marker (Cinnaclone, Tehran, Iran). The amplified PCR products of *mip* gene for each isolate were purified with the Gene JET™ Gel Extraction Kit (Fermentas, Lithuania) according to manufacturer's instructions. The sequences of the products were determined by Bioneer Company, Korea using an ABI PRISM 7700 Sequence Detection System (Applied Biosystems, Foster City, Calif. USA) according to the standard protocol of the supplier. The primers used for sequencing were as follows: forward primer (Legmip-fs), 5′-TTTATGAAGATGA(AG)A(CT)TGGTC(AG)CTGC-3′; and reverse primer (Legmip-r), 5′-TC(AG)TT(ATCG)GG(ATG)CC (ATG)AT(ATCG)GG(ATCG)CC(ATG)CC-3′.

### 2.6. Analysis of sequence data

The obtained *mip* gene sequences for each isolate were aligned separately and compared with all existing relevant sequences retrieved from GenBank database using the jPHYDIT program version 1. A percentage of similarity between the *mip* gene sequences of each isolate was determined by comparing sequences found to an in-house database of *mip* gene sequences (after data analysis by jPHYDIT program). The highest similarity percentage was considered as the identified species. For a confident identification, the similarity ≥98% to a sequence in the database was acceptable for submitted *mip* sequence.

## 3. Results

**Figure 1.**
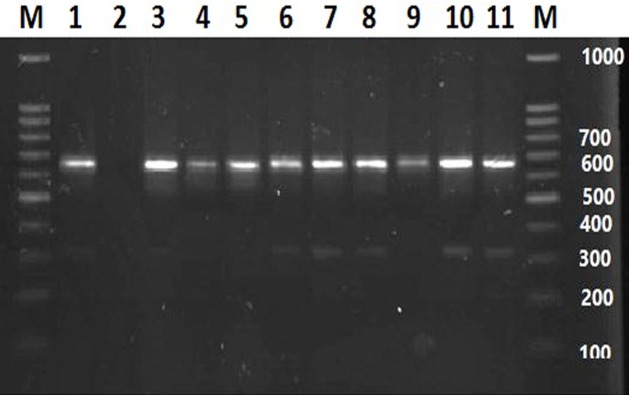
Electrophoresis of the PCR product following amplification of the *mip* gene (661–715 bp). Lane M: 100 bp DNA ladder; lane 1: Positive control (*L. pneumophila* ATCC33152); lane 2: Negative control (sterile distilled water); lanes 3–11, Positive samples.

Overall, 20 *Legionella* spp. were detected by culture method in 144 water samples (13.9%) in Ahvaz city, southwest Iran. All isolates were confirmed as *Legionella* spp. by *16S rRNA* gene PCR amplification. In this study, PCR of *mip* gene and its sequencing was used as reference identification method for detection of *Legionella* species (Figure 1). The water sources and distribution of identified *Legionella* species by *mip* sequencing are shown in Table 1. The result of *mip* sequencing and it`s analysis based on the hemology by jPHYDIT program revealed that L. pneumophila (with 13 isolates) was predominant (54.1%), whereas the Legionella species other than pneumophila, such as: L. *worsleinsis*, L. dumoffi, and L. fairfieldensis were accounted for 20.8%, 4.1% and 4.1%, respectively. In this study, the most common contaminated sources were hospital water baths (37 °C) with 34.6% and tap water with 25%, respectively (Table 2). However, no *Legionella* spp were detected in the water samples of neonatal incubator, nebulizer in ICU, hospital dialysis device, dental unit water, air conditioner, and drink water cooler.

**Table 1.** Identified *Legionella* and macrophage infectivity potentiator gene (*mip*) sequencing similarity percentage.

	Presumptive species	*mip* similarity percentage	Source
L1	*L .pneumophila*	98.93	Tap water
L2	*L .pneumophila*	100.00	Tap water
L3	*L .pneumophila*	100.00	Hospital water bath 37 °C
L4	*L .pneumophila*	100.00	Water filter system
L5	*L. worsleiensis*	99.47	Park fountain
L6	*L .pneumophila*	100.00	Hospital water bath 37 °C
L7	*L .pneumophila*	98.57	Park fountain
L8	*L .pneumophila*	98.39	Park fountain
L9	*L. worsleiensis*	98.75	Hospital water bath 37 °C
L10	*L .pneumophila*	98.57	Hospital water bath 37 °C
L11	*L. fairfieldensis*	100.00	Tap water
L12	*L .pneumophila*	100.00	Hospital Chiller
L13	*L .pneumophila*	100.00	Water tank reservoir
L14	*L .pneumophila*	100.00	Hospital water bath 37 °C
L15	*L .pneumophila*	99.82	Hospital water bath 37 °C
L16	*L .dumoffii*	100.00	Hospital water bath 37 °C
L17	*L. worsleiensis*	100.00	Hospital water bath 37 °C
L18	*L. worsleiensis*	100.00	Hospital water bath 37 °C
L19	*L .pneumophila*	100.00	Park fountain
L20	*L. worsleiensis*	100.00	Park fountain

**Table 2.** Occurrence of *Legionella* spp in different water samples.

	*Legionella* positivity(no. of positive/total no.)	*Legionella* distribution
Hospital water bath 37 °C	34.6% (9/26)	L3,L6,L9,L10,L14,L15,L16,L17,L18
Park fountain	20% (5/25)	L5,L7,L8,L19,L20
Water tank reservoir	7.1% (1/14)	L13
Hospital Chiller	16.6% (1/6)	L12
Neonatal incubator	0.0%(0/8)	-
Hospital nebulizer	0.0% (0/9)	-
Tap water	25.0% (3/12)	L1,L2,L11
Hospital dialysis device	0.0% (0/2)	-
Air conditioner	0.0% (0/3)	-
Water filter system	5.2% (1/19)	L4
Drink water cooler	0.0% (0/4)	-
Dental unit water	0.0% (0/16)	-
Total	13.9% (20/144)	

## 4. Discussion

Contamination of hospital water systems with *Legionella* spp. is a well-known cause of nosocomial Legionellosis. Inhalation of *Legionella* contaminated water aerosols could be cause of human infection. While the L. pneumophila is the main causative agent of legionellosis, the other species, such as L. micdadei, L. dumoffii, and L. bozemanii, are also known as other causes of disease in humans [18],[19]. The culture method is still known as a gold standard for *Legionella* detection, but it needs to a long time for isolation of *Legionella* species. In this study, we attempted to investigate different water sources of Ahvaz city, southwest Iran for presence of *Legionella* spp by culture. In addition, we used the mip sequencing method for the differentiation of *Legionella* spp.

Phylogenetic studies of *mip* gene were gradually introduced for their greater capacity than the 16S rRNA in discriminating between of *Legionella* species [15],[20]. In our study, some similarities which were observed in the *mip* gene amplicon among the 20 *Legionella* isolates, ranged from 98.57% to 100%. In this study, 13 isolated strains were identified as *L. pneumophila* (54.1%) from water samples by sequencing of *mip* gene. In consistence with our study, in a survey conducted on water systems in the southwest of Iran by culture and PCR methods, the prevalence of *L. pneumophila* was 41.1% [21]. In another study from Iran, 10 of 140 (7.1%) water samples were positive for *Legionella* species [22]. Our study confirmed the contamination of water sources of hospitals with the *Legionella* species that may play a key role as a risk factor for the patients' health in hospitals.

Moreover, it should be stressed that *L. pneumophila* is the most prevalent isolate in man-made aquatic environments, which should be seriously considered as a potential public health threat [23]–[25]. The past studies have been shown that the *mip* gene is a virulence factor which could provide the genetic evidence for high occurrence of *L. pneumophila* strains in man-made systems. This factor can resist to the intracellular killing against mammalian and protozoan phagocytic cells [26]. *Legionella* species, other than *L. pneumophila* also, may occur in man-made systems, however, more prevalence of *L. pneumophila* could be related to symbiotic interaction between this bacterium and free-living amoebae [27]. Furthermore, it seems that Legionella spp. other than *pneumophlia* with a lower infectivity and poor intracellular growth lead to more growth of virulent *L. pneumophila* strains in environmental sources [28].

The result of present study showed that *L. worsleiensis* (n = 5, 20.8%) was the second most observed isolate from the hospital water samples. Although the pathogenicity of *L. worsleiensis* is lower than L. pneumophila, however, a study has been claimed that presence of *L. worsleiensis* in environmental water samples (primarily in hospital water samples) might mask contamination with L. pneumophila, which could increase the infection risk with this organism [29]. The Legionella spp. could cause human pneumonia and accidentally induce other diseases, such as prosthetic valve endocarditis and septic arthritis [30],[31].

Similar to some studies, L. fairfieldensis and L. dumoffi were isolated from our hospital water samples. Svarrer *et al*. showed that these isolates together with Legionella micdadei were the most common cause of the culture-verified Legionella non-pneumophila infections in Denmark [29].

In another study by Stølhaug et al. all of 12 non-L. pneumophila reference strains were identified with a high accuracy by mip gene sequencing. This study also indicated that the mip gene sequencing could differentiate Legionella species [32].

## 5. Conclusions

This study highlights the need of continuous monitoring and risk assessment of water supplies of hospitals in our region for *Legionella* spp. contamination. Furthermore, despite the fact that hospitals use a purified water system, 13.9% of the samples were positive for *Legionella* spp. Therefore, it can be concluded that the common methods of water purification and disinfection are not sufficient for purification of the water network from these bacteria and new policies should be put in place to control and eliminate them in water resources. Also, it is recommended that using the PCR method along with the *mip* gene sequencing could detect non-pneumophila Legionella species in routine surveillance of Legionella in water samples.

## Limitation in this study

Although a neutralizing agent is needed in order to neutralize residual disinfectants, however no use of such agent in present study was identified as a limiting factor.
